# Miscellaneous

**Published:** 1876-08

**Authors:** 


### Miscellaneous.

### THE LEAD LINE OF THE GUMS.

Some further facts were communicated last month to the Royal Medical and Chirurgical Society, in a paper by Dr. C. Hilton Fagge:—

The author had in two cases investigated the microscopical characters of the gingival tissues that were affected with the lead line. He found that the discolorations were not uniform, but were distributed in the form of rounded loops, corresponding with the vascular papillæ of the mucous membrane. Under a higher power the pigment is seen to consist of granules, some of which were deposited in the interior of the smallest blood vessels, others immediately outside of them. Mr. Tomes' hypothesis was that the pigment was a sulphuret of lead; and that the sulphuretted hydrogen which formed it was derived from decomposing animal matters which had been retained in the tartar or between the teeth. The author pointed out that the gas must diffuse itself into the textures of the gum and combine with the lead, as it was actually circulating in the blood. This view explained the fact that a characteristic line in the gums was sometimes seen in those who had not shown symptoms of lead poisoning. When occurring under such circumstances, it had been supposed by certain writers to have been caused by some other metal, such as bismuth. The author thought that it depended on the very minute quantity of lead which had in such cases gained entrance into the body. Another point explicable upon the author's theory of the lead line was, that the administration of iodide of potassium sometimes brought it out, when it had before been absent, and after the individual had ceased to be exposed to the poison. It also followed that the line must be wanting in those who keep the teeth very clean. Lastly it seemed probable that the sulphide of lead must be deposited in the intestinal mucous membrane likewise under the influence of the sulphuretted hydrogen contained in the bowels.

### STAMMERING A HEREDITARY DEFECT.

Dr. Coen in the *Wiener Med. Zeitung*, states that Schulthess, Colombat, Rosenthal, and others, have amply proved that stammering may be a family defect (especially on the mother's side), and may also be acquired through voluntary or involuntary imitation. Speaking of his own experience, Dr. Coen states that among fifty carefully observed cases, he found that in nine the stammering was due to the above causes, viz.: in five to heredity, and in four to imitation. According to his observation, confirmed also by the statement of Colombat, stammering thus produced is of a less favorable prognosis, while its treatment is more difficult and more prolonged, so that recovery is only to be expected when the cases are treated with unusual energy. A rational prophylaxis is of the greatest importance in children inclined to stammer, and details on this will be given in the forthcoming work.

In illustration, Dr. Coen relates one of the five hereditary cases he met with. A boy ten years old, was brought to him with an excessive stammer, having always been on the increase from the commencement. His mother stammered until her eighteenth year, and now, in her thirty-seventh year, she does not speak quite freely. Her parents were free from stammer but among their five children a brother as well as herself suffered from it. Moreover the mother of the boy had an aunt (forty-two years of age) and two uncles (forty-six and fifty-seven years of age) who both stammered from their youth. The mother of an aunt of this woman also stammered. Both the boy and his mother as well as his sister, were scrofulous, whereas the father had died in a good state of health, and did not stammer. Here therefore was a striking example of the heredity of stammering, in which the affection could be traced through three generations, affecting several members of the family. It is also of importance to state that it arose on the mother's side.

### ELECTROLYSIS IN ANEURISM.

At the close of a lecture, reported in the *British Medical Journal*, Mr. J. Duncan, lecturer on surgery in the University of Edinburgh, said:—

I shall now endeavor to sum up for you the conclusions which I draw from a review of the history of electrolysis in aneurism, and my own experience of its workings. I beg you to understand that I speak tentatively only; that I am not inclined to dogmatize.

1. In aortic aneurism, electrolysis is available to prevent external hemorrhage; and in such cases the operation should be prolonged, that it may occupy, so far as is possible, the external sac with the electrolytic products.

2. Even when the aneurism has not penetrated the wall of the thorax or abdomen, electrolysis may be hopefully used after a trial of other remedies, but should not be pushed to the same extent.

3. In external aneurisms, electrolysis should be tried, if compression be not available, or have failed: and it is to be preferred to ligature—at least of the innominate and iliac arteries.

4. Both poles ought to be inserted, unless the aneurism be fusiform, or too small to admit them.

5. If only one pole be used, the positive should be inserted, because its clot is firmer and more adherent though smaller.

6. The needles ought to be insulated, by means of vulcanite.

7. The battery should have large electromotor force. If both poles be inserted, from four to six cells of medium size should be used; if one only, a large number of cells of smaller size are required.

